# Association of glucagon-like peptide-1 receptor agonists with suicidal ideation and self-injury in individuals with diabetes and obesity: a propensity-weighted, population-based cohort study

**DOI:** 10.1007/s00125-024-06243-z

**Published:** 2024-08-06

**Authors:** Isabel Hurtado, Celia Robles, Salvador Peiró, Aníbal García-Sempere, Gabriel Sanfélix-Gimeno

**Affiliations:** 1 Health Services Research & Pharmacoepidemiology Unit, Foundation for the Promotion of Health and Biomedical Research of Valencia Region, Fisabio, Valencia, Spain; 2 Spanish Network for Research on Chronicity, Primary Care, and Health Promotion (RICAPPS), Valencia, Spain

**Keywords:** Cohort study, Diabetes, GLP-1RA, Glucose-lowering drugs, Obesity, Self-injury, SGLT-2i, Suicidal ideation

## Abstract

**Aims/hypothesis:**

Regulators worldwide are reviewing safety data on glucagon-like peptide-1 receptor agonists (GLP-1RA), following reports by the Icelandic Medicines Agency in July 2023 of suicidal ideation and self-injury (SIS) in individuals taking liraglutide and semaglutide. We aimed to assess the risk of SIS in new users of GLP-1RA when compared with sodium-glucose cotransporter 2 inhibitors (SGLT-2i) users, prescribed to treat type 2 diabetes in individuals with obesity.

**Methods:**

This is a cohort study combining several population-wide databases and covering a Spanish population of five million inhabitants, including all adults with obesity who initiated treatment with either GLP-1RA or SGLT-2i for type 2 diabetes from 2015 to 2021. To estimate the comparative effect of GLP-1RA on the risk of SIS, we employed a new user, active comparator design and we carried out multivariable Cox regression modelling with inverse probability of treatment weighting (IPTW) based on propensity scores. We performed several stratified and sensitivity analyses.

**Results:**

We included 3040 patients initiating treatment with GLP-1RA and 11,627 with SGLT-2i. When compared with patients treated with SGLT-2i, those in the GLP-1RA group were younger (55 vs 60 years old, *p*<0.001), had more anxiety (49.4% vs 41.5%, *p*<0.001), sleep disorders (43.2% vs 34.1%, *p*<0.001) and depression (24.4% vs 19.0%, *p*<0.001), and were more obese (35.1% of individuals with BMI ≥40 vs 15.1%, *p*<0.001). After propensity score weighting, standardised mean differences between groups were <0.1 for all covariates, showing adequate balance between groups at baseline after adjustment. In the main per-protocol analyses we found no evidence that GLP-1RA increased the incidence of SIS (HR 1.04; 95% CI 0.35, 3.14). Intention-to-treat analyses resulted in an HR of 1.36 (95% CI 0.51, 3.61). In analyses excluding individuals with no BMI information and using imputation for BMI missing values, respective HRs were 0.89 (95% CI 0.26, 3.14) and 1.29 (95% CI 0.42, 3.92). Stratified analyses showed no differences between subgroups.

**Conclusions/interpretation:**

Our findings do not support an increased risk of SIS when taking GLP-1RA in individuals with type 2 diabetes and obesity; however, the rarity of SIS events and the wide uncertainty of effect size (although null, effect may be compatible with a risk as high as threefold) calls for a cautious interpretation of our results. Further studies, including final evaluations from regulatory bodies, are called for to discard a causal link between GLP-1RA and suicidality.

**Graphical Abstract:**

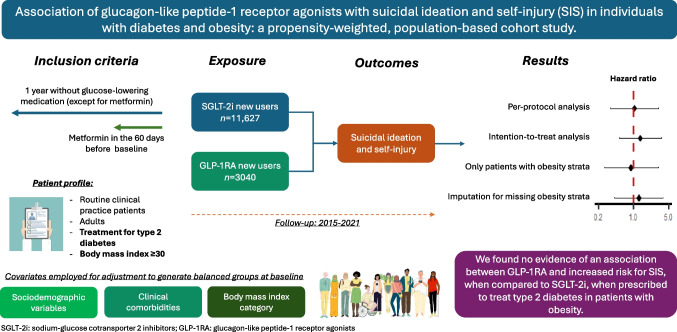

**Supplementary Information:**

The online version of this article (10.1007/s00125-024-06243-z) contains peer-reviewed but unedited supplementary material.


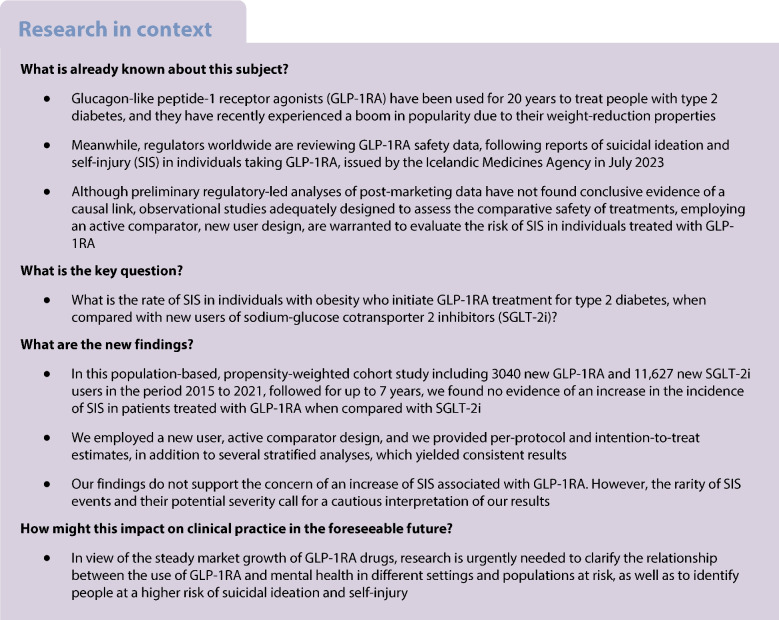


## Introduction

In the last 20 years, glucagon-like peptide-1 receptor agonists (GLP-1RA) have been used as one of the recommended pharmacotherapy options for individuals with type 2 diabetes mellitus, especially for large subgroups of patients with high cardiovascular risk and other comorbidities such as chronic kidney disease [1]. In several studies, GLP-1RA have been proved to reduce glucose levels, to ameliorate the metabolic syndrome and to provide cardiorenal benefits [2–5]. Furthermore, GLP-1RA have shown substantial weight-reduction effects [6], and in recent years several compounds have been authorised for weight management in people with overweight or obesity. Since then, GLP-1RA have received considerable media and influencer attention which, combined with intensive commercial practices, have led to a popularity storm and an explosion in the use of these drugs, resulting in global shortages of GLP-1RA drugs [7–9].

In contrast, there is a growing concern that GLP-1RA may be linked to increased risk of suicidal ideation and self-injury (SIS), following reports of the Icelandic Medicines Agency in July 2023 identifying a disproportionate number of cases of SIS in individuals taking liraglutide and semaglutide. Previously, other weight-loss drugs have been removed from the market for similar reasons [10], and regulators worldwide are currently reviewing safety data on GLP-1RA [11–13]. Although preliminary regulatory-led analyses of post-marketing data have not found conclusive evidence of a causal link [14], and in the absence of evidence from large, randomised clinical trials, observational studies adequately designed to assess the comparative safety of treatments, employing an active comparator, new user design, are warranted to evaluate the risk of SIS in individuals treated with GLP-1RA.

In this study, we aimed to assess the risk of SIS in new users of GLP-1RA to treat type 2 diabetes mellitus in individuals with obesity, when compared with new users of sodium-glucose cotransporter 2 inhibitors (SGLT-2i).

## Methods

### Design, setting and data sources

This is a real-world cohort study combining several population-wide databases from the Valencia Health System Integrated Database (VID). The Valencia Health System (VHS) is a comprehensive structure of hospitals, primary care facilities and other public resources managed by the government of the region of Valencia in Spain providing free, universal healthcare services (besides drug cost‐sharing) to 98% of the region's five million inhabitants. VID is a set of publicly owned, population-based healthcare, clinical and administrative electronic databases in the region that can be linked by means of a single personal identification number, and provides comprehensive information for the region’s population covered by the VHS since 2008. VID includes sociodemographic and administrative data (sex assigned at birth, age, nationality) as well as healthcare information such as inpatient and outpatient diagnoses, procedures, laboratory data, pharmaceutical prescriptions and dispensing linked at the individual level (including brand and generic name, formulation, strength and dosing schedule/regimen), hospitalisations, primary care electronic medical record data, mortality, healthcare utilisation and public health data [15].

### Participants

**Fig. 1:**
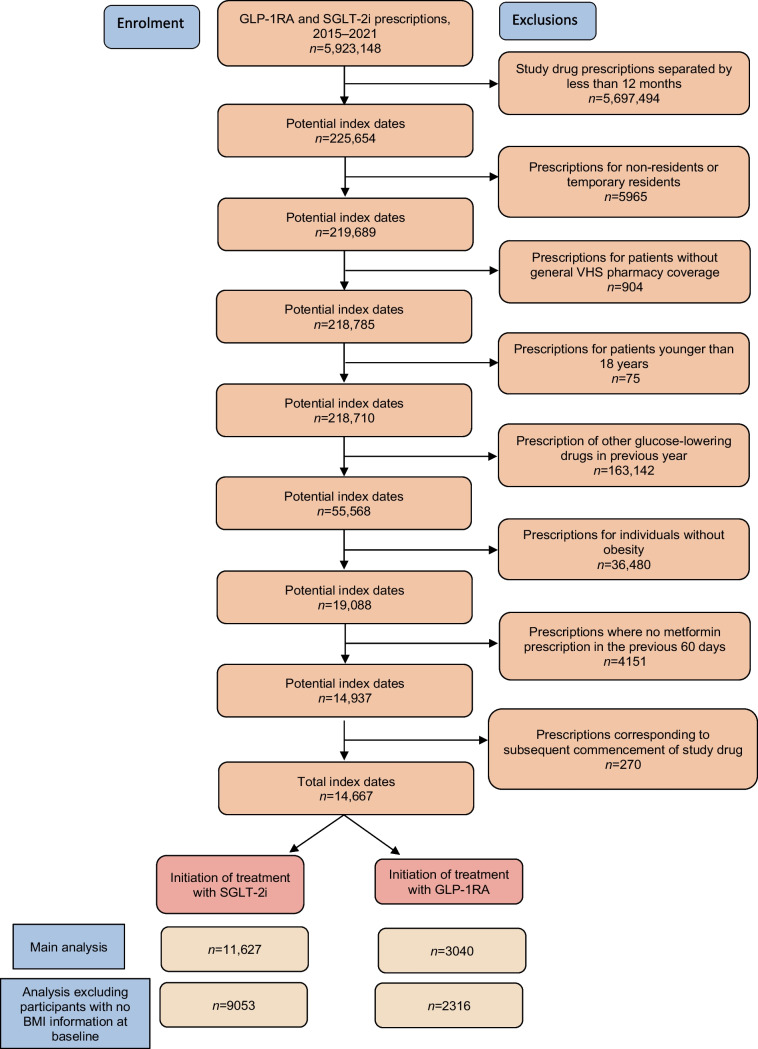
Study flow chart

We included all individuals aged 18 years and over who initiated either GLP-1RA or SGLT-2i treatment for type 2 diabetes from 1 January 2015 to 31 December 2021. Patients filling a prescription for either GLP-1RA or SGLT-2i medication and not having filled a prescription for a glucose-lowering drug (except for metformin) in the previous 365 days were considered new users. Taking into account that SGLT-2i and GLP-1RA are recommended as therapeutic alternatives by many clinical guidelines, usually as second-line treatments after first-line metformin, new users of SGLT-2i appear to be a suitable active comparator group. The index date was the day of first refill of a study drug. Participants were required to have obesity at baseline, defined as BMI ≥30, or a diagnosis of obesity using International Classification of Diseases, Ninth and Tenth Revision (ICD-9 [http://www.icd9data.com/2007/Volume1/default.htm] and ICD-10 [https://icd.who.int/browse10/2019/en]), registered in the VID medical record in the 365 days before the index date, and to be taking metformin on the index date. GLP-1RA are only authorised for and used in the study setting in individuals with a BMI ≥30; their prescription is subject to a prior authorisation procedure, compelling the prescriber to have its prescription validated—by a medical inspector who verifies that the prescription is compliant with the authorised indication—before it is accepted for public funding and dispensing [16]. In this way, we only included new SGLT-2i or GLP-1RA users with obesity when initiating therapy, in order to produce groups as similar as possible at baseline. Use of metformin in the index date was defined by having a prescription for metformin issued in the 60 days before the index date. Finally, people without general VHS pharmacy coverage (mainly certain Spanish government employees whose prescriptions are reimbursed by mutual societies for civil servants and are thus not included in the pharmacy databases of the VHS), and people not registered in the municipal census (non-residents or temporary residents), were excluded because of limitations on follow-up (see Fig. 1 flow chart).

### Variables

We included the following patient sociodemographic, lifestyle and clinical variables (see Table 1): age (18 to 44 years; 45 to 64; 65 to 74; 75 and over); sex assigned at birth; income level (retrieved from the pharmaceutical copayment system that establishes different levels of copayment based on the following income categories: people earning less than €18,000/year, €18,000 to €100,000/year, more than €100,000/year, population exempt from copayment due to socioeconomic vulnerability (labelled in our study as the ‘low resources’ group), and people without income data (labelled as ‘not available’); registered tobacco and alcohol use in VID in the 365 days preceding the index date; active comorbidities in VID at baseline (diagnostic codes in the VID electronic medical record may be activated at the onset of a disease and deactivated after; typically diagnostic codes for chronic conditions remain active for life), including several psychiatric comorbidities (heart failure, dementia, hypertension, liver disease, renal disease, coronary heart disease, congestive obstructive pulmonary disease, malignancies, depression, sleep disorders, anxiety, substance abuse, personality disorders, psychotic disorders, other psychiatric disorders), previous suicidal ideation or self-injury; and BMI at baseline (30–35, 35–40, and ≥40 kg/m^2^). See electronic supplementary material (ESM) Table 1 for ICD, and ESM Table 2 for Anatomical Therapeutic Chemical (ATC) codes used for covariates and drugs included in the study.

**Table 1:** Participant characteristics at baseline, overall and for SGLT-2i and GLP-1RA new users

Characteristic	Total, *N* (%)	SGLT-2i, *n* (%)	GLP-1 RA,* n* (%)	Stand. diff. before	Stand. diff. after	*p* value
	14,667	11,627 (79.27)	3040 (20.73)			
Sex, men	7877 (53.71)	6491 (55.83)	1386 (45.59)	0.206	0.006	<0.001
Income, €/year						
<18,000	9489 (64.70)	7636 (65.67)	1853 (60.95)	−0.098	−0.031	<0.001
18,000–100,000	3054 (20.82)	2337 (20.10)	717 (23.59)	0.084	0.022	
>100,000	49 (0.33)	33 (0.28)	16 (0.53)	0.038	0.008	
Low resources	1970 (13.43)	1531 (13.17)	439 (14.44)	0.037	0.015	
Not available	105 (0.72)	90 (0.77)	15 (0.49)	−0.035	0.003	
Age, years						
18–44	1787 (12.18)	1174 (10.10)	613 (20.16)	0.284	0.004	<0.001
45–64	8064 (54.98)	6262 (53.86)	1802 (59.28)	0.109	0.007	
65–74	3516 (23.97)	3006 (25.85)	510 (16.78)	−0.223	−0.009	
≥75	1300 (8.86)	1185 (10.19)	115 (3.78)	−0.253	−0.003	
Comorbidities						
Heart failure	1067 (7.27)	908 (7.81)	159 (5.23)	−0.105	−0.026	<0.001
Dementia	278 (1.90)	234 (2.01)	44 (1.45)	−0.043	−0.017	0.05
Hypertension	10,440 (71.18)	8376 (72.04)	2064 (67.89)	−0.090	−0.016	<0.001
Liver disease	3346 (22.81)	2626 (22.59)	720 (23.68)	0.026	0.007	0.207
Renal disease	805 (5.49)	656 (5.64)	149 (4.90)	−0.033	0.014	0.121
Depression	2950 (20.11)	2207 (18.98)	743 (24.44)	0.133	−0.003	<0.001
Coronary heart disease	1701 (11.60)	1465 (12.60)	236 (7.76)	−0.160	0.006	<0.001
COPD	1501 (10.23)	1217 (10.47)	284 (9.34)	−0.038	0.007	0.074
Sleep disorders	5276 (35.97)	3962 (34.08)	1314 (43.22)	0.189	0.002	<0.001
Anxiety	6324 (43.12)	4821 (41.46)	1503 (49.44)	0.161	0.021	<0.001
Substance abuse	221 (1.51)	158 (1.36)	63 (2.07)	0.055	−0.003	0.005
Personality disorders	200 (1.36)	140 (1.20)	60 (1.97)	0.062	−0.008	0.002
Other psychiatric disorders	598 (4.08)	431 (3.71)	167 (5.49)	0.085	−0.008	<0.001
Psychotic disorders	634 (4.32)	480 (4.13)	154 (5.07)	0.045	−0.002	0.027
Previous SIS	29 (0.20)	25 (0.22)	4 (0.13)	−0.020	−0.003	0.488
Malignancies	1503 (10.25)	1221 (10.50)	282 (9.28)	−0.041	0.016	0.051
Lifestyle						
Alcohol use	656 (4.47)	547 (4.70)	109 (3.59)	−0.056	0.024	0.009
Tobacco use	3474 (23.69)	2740 (23.57)	734 (24.14)	0.014	0.012	0.519
BMI						
30–35	5148 (35.10)	4604 (39.60)	544 (17.89)	−0.494	−0.007	<0.001
35–40	3394 (23.14)	2689 (23.13)	705 (23.19)	0.002	0.011	
≥40	2827 (19.27)	1760 (15.14)	1067 (35.10)	0.473	0.014	
Unknown^a^	3298 (22.49)	2574 (22.14)	724 (23.82)	0.040	−0.017	

Data are *n* (%)

Standardised differences before and after IPTW are shown for variables included in the Cox models

^a^Unknown BMI: patients classified as obese based on a registered ICD code for obesity without specifying BMI level

COPD, chronic obstructive pulmonary disease; stand. diff. after, standardised difference after IPTW; stand. diff. before, standardised difference before IPTW

### Outcomes

Primary outcomes were SIS. Outcomes were retrieved from different real-world databases in VID (primary care electronic records, in-hospital and outpatient hospital records and emergency room records), via ICD codes registered during healthcare encounters (see ESM Table 1). In the main per-protocol analyses, participants were followed from the index date to first mutually exclusive incidence of SIS, discontinuation of study drug (defined as more than 60 days without drug supply), initiation of other study drug, or switch to other glucose-lowering drug (defined as filling a prescription for any glucose-lowering drug other than the baseline treatment or metformin and not refilling the assigned drug for 60 days), death, or end of follow-up. In secondary, intention-to-treat analyses, participants were followed from the index date to first SIS event, death, or end of follow-up, and were analysed according to the treatment assigned at baseline, irrespective of actual exposure during follow-up.

### Analysis

First, we described patient characteristics, overall and for GLP-1RA and SGLT-2i users (see Table 1). The *p* values were estimated using *χ*^2^ tests for categorical variables and *t* tests for continuous variables. Second, as the main analysis, we used a per-protocol approach to estimate the increased risk of SIS in GLP-1RA vs SGLT-2i users. The per-protocol approach evaluates the comparative effect of actual exposure to the treatment strategies assigned at baseline. We estimated the incidence rates by 1000 person-years of the first SIS event, overall and for each group. Second, we carried out multivariable Cox regression modelling using our set of covariates: age (as a categorical variable, reference [ref.] 18 to 44 years old), sex assigned at birth (ref. men), all comorbidities and lifestyle variables included as categorical variables (yes/no), and BMI (ref. 30–35 kg/m^2^). To adjust for potential confounding, we used inverse probability of treatment weighting (IPTW) based on propensity scores, a method that allows balancing without losing generalisability [17]. Propensity scores for each outcome were calculated based on the probability of initiating treatment with GLP-1RA taking into account the same covariates, to generate patient-specific stabilised weights. Covariate balance between the weighted exposure cohorts was assessed using standardised mean differences, with standardised differences <0.10 suggesting adequate balance [18]. Third, we performed several sensitivity analyses. We provided an intention-to-treat analysis, where we compared the effect of being assigned to the treatment strategies at baseline, regardless of whether or not the individuals continued to follow the strategies during follow-up. We also conducted several per-protocol stratified analyses: by the more prevalent psychiatric comorbidities in our cohort (depression, anxiety and sleep disorders); by sex; and by obesity levels; and we provided *p* values for interaction. Finally, to deal with participants with no BMI information at baseline, we also carried out analyses excluding individuals with no BMI information, in addition to an analysis using multivariate imputation using chained equations for BMI missing values (see ESM Table 3 for code employed for analysis). Statistical significance was defined as *p*<0.05. Ethics approval was obtained from the Clinic University Hospital of Valencia research ethics board (2022/164) with a waiver of informed consent. All analyses were performed using STATA version 14 and R version 3.6.0.). The investigators were granted access to the databases used to create the study population and performed several quality checks (consistency assessments, range and logic checks) before the linkage of the databases using a pseudonymised single identification number. The study was reported using the RECORD checklist for observational studies using routinely collected health data.

## Results

We included 3040 individuals initiating treatment with GLP-1RA and 11,627 with SGLT-2i (Fig. 1). Mean age was 59 years, 53.7% were men, 64.7% earned less than €18,000/year, 71.2% had hypertension, 43.1% had anxiety, 36.0% had sleep disorders and 23.7% had registered tobacco use (Table 1). When compared with participants initiating treatment with SGLT-2i, those in the GLP-1RA group were younger (55 vs 60 years, *p*<0.001), were more likely to be women (54.4% vs 44.2%, *p*<0.001), had more anxiety (49.4% vs 41.5%, *p*<0.001), sleep disorders (43.2% vs 34.1%, *p*<0.001) and depression (24.4% vs 19.0%, *p*<0.001), and were more obese (35.1% of individuals with BMI ≥40 vs 15.1%, *p*<0.001). After propensity score weighting, standardised mean differences between groups were <0.1 for all covariates, showing adequate balance between groups at baseline after adjustment (Table 1).

Weighted incidence rates were similar in both groups (Table 2). In the main per-protocol analyses, mean follow-up was 483 days in the GLP-1RA and 397 days in the SGLT-2i group. We found no evidence that GLP-1RA increased the incidence of SIS (HR 1.04; 95% CI 0.35, 3.14). Intention-to-treat analyses (mean follow-up: 970 days for SGLT-2i users and 992 days for GLP-1RA users) resulted in an HR of 1.36 (95% CI 0.51, 3.61). In analyses excluding individuals with no BMI information and using multiple imputation for BMI missing values, respective HRs were 0.89 (95% CI 0.26, 3.14) and 1.29 (95% CI 0.42, 3.92) (Table 2). Stratified analyses showed no differences in the risk of SIS when prescribed GLP-1RA or SGLT-2i between participants with and without depression, anxiety or sleep disorders, between women and men, and between individuals in different obesity categories (ESM Fig. 1).

**Table 2:** Risk of suicidal ideation or self-injury among new users of GLP-1RA vs SGLT-2i

		Person-years	Crude outcomes	Crude rate/1000 person-years	Weighted outcomes	Weighted rate/1000 person-years (95% CI)	HR (95% CI)
Main analysis	SGLT-2i	12,575.93	17	1.35	18.11	1.44 (0.88, 2.52)	Ref.
	GLP-1RA	3785.42	10	2.64	6.66	1.76 (0.88, 4.09)	1.04 (0.35, 3.14)
Intention-to-treat analysis	SGLT-2i	31,823.39	17	0.53	18.11	0.57 (0.35, 1.00)	Ref.
	GLP-1RA	7562.30	10	1.32	6.66	0.88 (0.44, 2.05)	1.36 (0.51, 3.61)
Analysis excluding patients with BMI missing data	SGLT-2i	9966.08	13	1.30	14.76	1.48 (0.83, 2.93)	Ref.
	GLP-1RA	2714.90	7	2.58	4.54	1.67 (0.71, 4.95)	0.89 (0.26, 3.14)
Analysis with multiple imputation for BMI missing data	SGLT-2i	12,575.93	17	1.35	18.11	1.44 (0.88, 2.52)	Ref.
	GLP-1RA	3785.42	10	2.64	6.66	1.76 (0.88, 4.09)	1.29 (0.42, 3.92)

Ref., reference

## Discussion

In this population-based, propensity-weighted cohort study, we found no evidence of an association between GLP-1RA and increased risk for SIS when compared with SGLT-2i, when prescribed to treat type 2 diabetes in patients with concomitant obesity. Our findings do not support an increased risk of SIS associated with GLP-1RA. All 95% CIs were compatible with no increase in risk. However, CIs of the observed associations are wide because of the small number of events. As a result of this, protective or adverse effects cannot be fully excluded. Because of the low frequency of SIS outcomes and their potential severity, and based on our estimates, we cannot rule out either a threefold increase or a decrease in SIS rates among people with type 2 diabetes and obesity treated with GLP-1RA. Large trials randomising tens of thousands of patients would be required to accurately detect an effect of GLP-1RA on SIS risk; in the absence of such trials, evidence from high quality pharmacoepidemiological studies employing population-based, observational data, such as this study, is warranted.

GLP-1RA are indicated for diabetes and obesity, which in turn are concomitant risk factors for depression and SIS. Available evidence, however, is difficult to interpret. For instance, a reciprocal association between obesity and overweight and depression has been observed [19], whereas in most studies elevated BMI is consistently associated with lower suicide completion [20]. Whether obesity is associated with suicide attempts or ideation remains unclear, as only a few, methodologically flawed studies have assessed these associations, providing heterogeneous findings, both between studies and in key risk groups [21]. Plausible mechanisms have been proposed that could explain an association of GLP-1RA with suicidality; other authors support a potential anti-depressant effect of GLP-1RA, others a lack of association. For instance, GLP-1RA have been tested in people with serious mental illness, who could be at a high suicidality risk, showing metabolic benefits with no increase in psychopathology or occurrence of SIS [22].

To date, evidence for an association between GLP-1RA and SIS is limited to a few preliminary reports from pharmacovigilance systems and pharmacoepidemiological studies. Randomised controlled trials involving GLP-1RA have not detected suicidality signals, but these studies were underpowered to detect rare events such as SIS, and they selectively exclude individuals with psychiatric comorbidity [23–26], which are prevalent in daily practice. Studies from the US Food and Drug Administration (FDA) have found a disproportionate reporting of suicidal ideation and ‘depression/suicidal’ thoughts with semaglutide and liraglutide, but not for suicidal behaviour, suicide attempts and completed suicide [27–30]. Reports from the European Medicines Agency (EMA) were due in November 2023, but the EMA asked for more data from manufacturers in December 2023, and final results are still unreleased [11, 31]. To date, only one study into this subject has been published, with more suicidal events being reported associated with semaglutide and liraglutide when compared to other GLP-1RA [32]. However, pharmacovigilance reporting in public databases is subject to many major limitations, such as heterogeneous reporting, including notoriety and ripple bias, double reporting, the lack of a population denominator or enough clinical detail to enable comparison between agents and over time [20, 22], therefore no causal association link can be inferred from such studies.

Two propensity score matched observational studies have been conducted to date, either evaluating suicidality risk in patients using semaglutide vs non-GLP-1RA glucose-lowering agents, or in those using any GLP-1RA vs users of dipeptidyl peptidase-4 (DPP-4) inhibitors. Both studies found a protective effect of GLP-1RA, which was consistent in several analyses for different indications (obesity or diabetes) and patient profiles (with or without previous depression or suicidal ideation) [33, 34]. In this way, available observational evidence to date refutes concerns of an increase in suicidality risk related to GLP-1RA treatment. However, both studies are based on a large but not representative sample of the US population, and generalisability of results is limited. Also, both provide intention-to-treat estimates only, and were unable to evaluate either medication adherence or the effect of actual exposure to GLP-1RA [33]. Our study adds to the scarce available evidence on the association between GLP-1RA and risk of SIS by providing evidence from a South European population of five million inhabitants. We employed a new user, active comparator design; we chose a suitable comparator group; we employed propensity scores based on IPTW to obtain balanced pseudo-populations at baseline; we provided both per-protocol and intention-to-treat estimates; and we performed several stratified analyses. In all analyses including stratified analyses per sex assigned at birth, we did not detect an association between SIS risk and GLP-1RA when compared with SGLT-2i. Our findings do not support an increased risk of SIS when taking GLP-1RA; however, rarity of SIS events calls for a cautious interpretation of our results.

Our study is subject to some limitations. First, the VID databases gather several sources of real-world clinical practice data and contain information as registered by health professionals during routine clinical practice, but data are not specifically prepared for research. In this sense, studies based on real-world clinical information like the VID are at risk of well-known biases such as differential recording, misclassification bias or missing data. For instance, identification of suicidal outcomes by means of ICD codes registered during routine clinical practice databases has been associated with a slight under-registration of cases, which may result in underestimation of the association between drug exposure and SIS outcomes [35], and studies validating suicidal classification have concluded that ICD codes have good positive predictive value but low specificity [36]. Second, despite accounting for many relevant covariates in the adjustment and employing IPTW techniques, we may have missed some potentially relevant information and thus we cannot rule out residual confounding. For instance, covariates such as income or BMI strata included categories for missing data, which may introduce bias in observational studies. However, results of sensitivity analyses were, overall, comparable to those of our main analysis, suggesting robustness of the observed associations. Also, given the observational nature of the study, we could expect the presence of indication bias, a type of confounding bias that occurs when a symptom or sign of disease is judged as an indication for a given therapy, and is therefore associated both with the use of a drug and with a higher probability of an outcome related to the disease for which the agent is indicated [37]. In this sense, our results must be interpreted with caution. Third, suicidal outcomes are rare events; because of the low incidence of SIS, and based on our findings, it is not possible to rule out either a threefold increase or a decrease in its occurrence among people treated with GLP-1RA. Therefore, association estimates should be interpreted with extreme caution. Fourth, coding decision-making in real-world practice may be subject to variability, a potential bias frequent in real-world studies. Fifth, information on inpatient medication is not available in VID, which may result in marginal misestimation. Sixth, stratified analyses resulted in some groups with a relatively low number of participants and events, where risk estimates should be considered as exploratory findings. Finally, the generalisation of our results to other settings outside Spain, or even to other Spanish regions, should be approached with caution. However, even if findings in one population cannot be generalised to a new setting if the association of interest is modified by patient characteristics, settings, or treatment variations which differ in the new setting, our findings are comparable to those observed in pharmacovigilance and pharmacoepidemiologic studies that do not find increased risk of SIS associated with GLP-1RA.

In view of the steady market growth of GLP-1RA drugs and prospects of potential expansion to hugely profitable and self-selling indications such as weight management or Parkinson’s disease [38], research is urgently needed to clarify the relationship between the use of GLP-1RA and mental health, as well as to identify individuals at a higher risk of suicidal ideation and self-injury. Further studies, including final evaluations from regulatory bodies, are warranted to discard a causal link between GLP-1RA and suicidality (shortly before publication of this article, EMA released a news report in line with our findings [39]).

## Supplementary Information

Below is the link to the electronic supplementary material.

ESM 1 (PDF 258 KB)

## Data Availability

The datasets presented in this article are not readily available due to legal restrictions on sharing the dataset as regulated by the Valencia regional government by means of legal resolution by the Valencia Health Agency [2009/13312] which forbids the dissemination of data to third parties (accessible at: https://www.san.gva.es/documents/d/investigacio/resolucion_solicitud_datos_es_va). Upon request, authors can allow access to the databases to verify the accuracy of the analysis or the reproducibility of the study. Requests to access the datasets should be directed to the Management Office of the Data Commission in the Valencia Health Agency (email: solicitud_datos@gva.es; telephone numbers: +34 961-928207; +34 961-928198).

## Abbreviations

GLP-1RA: Glucagon-like peptide-1 receptor agonist(s)
IPTW: Inverse probability of treatment weighting
SGLT-2i: Sodium-glucose cotransporter 2 inhibitor(s)
SIS: Suicidal ideation and self-injury
VHS: Valencia Health System
VID: Valencia Health System Integrated Database

## References

[1] Davies MJ, Aroda VR, Collins BS et al (2022) Management of hyperglycemia in type 2 diabetes, a consensus report by the American Diabetes Association (ADA) and the European Association for the Study of Diabetes (EASD). Diabetes Care 45(11):2753–2786. 10.2337/dci22-0034 36148880 10.2337/dci22-0034 PMC10008140

[2] Lingvay I, Leiter LA (2018) Use of GLP-1 RAs in cardiovascular disease prevention: a practical guide. Circulation 137(21):2200–2202. 10.1161/CIRCULATIONAHA.117.032759 29784677 10.1161/CIRCULATIONAHA.117.032759

[3] Patorno E, Htoo PT, Glynn RJ et al (2021) Sodium-glucose cotransporter-2 inhibitors versus glucagon-like peptide-1 receptor agonists and the risk for cardiovascular outcomes in routine care patients with diabetes across categories of cardiovascular disease. Ann Intern Med 174(11):1528–1541. 10.7326/M21-0893 34570599 10.7326/M21-0893 PMC8969214

[4] Ma X, Liu Z, Ilyas I et al (2021) GLP-1 receptor agonists (GLP-1RAs): cardiovascular actions and therapeutic potential. Int J Biol 17(8):2050–2068. 10.7150/ijbs.59965 10.7150/ijbs.59965 PMC8193264 34131405

[5] Barritt AS, Marshman E, Noureddin M (2022) Review article: role of glucagon-like peptide-1 receptor agonists in non-alcoholic steatohepatitis, obesity and diabetes-what hepatologists need to know. Aliment Pharmacol 55(8):944–959. 10.1111/apt.16794 10.1111/apt.16794 PMC9310586 35266164

[6] Jastreboff AM, Kushner RF (2023) New frontiers in obesity treatment: GLP-1 and nascent nutrient-stimulated hormone-based therapeutics. Annu Rev Med 74:125–139. 10.1146/annurev-med-043021-014919 36706749 10.1146/annurev-med-043021-014919

[7] Blum D (2022) What is Ozempic and why is it getting so much attention? Available from https://www.nytimes.com/2022/11/22/well/ozempic-diabetes-weight-loss.html. Accessed 5 April 2024

[8] Johnson A (2023) What to know about Ozempic: the diabetes drug becomes a viral weight loss hit (Elon Musk boasts using it) creating a shortage. Available from https://www.forbes.com/sites/ariannajohnson/2022/12/26/what-to-know-about-ozempic/?sh=6bf068045adb. Accessed 5 April 2024

[9] World Health Organization (2024) Shortages impacting access to glucagon-like peptide 1 receptor agonist products; increasing the potential for falsified versions. Available from https://www.who.int/news/item/29-01-2024-shortages-impacting-access-to-glucagon-like-peptide-1-receptor-agonist-products--increasing-the-potential-for-falsified-versions. Accessed 5 April 2024

[10] Sam AH, Salem V, Ghatei MA (2011) Rimonabant: from RIO to ban. J Obes 2011:432607. 10.1155/2011/432607 21773005 10.1155/2011/432607 PMC3136184

[11] European Medicines Agency (2024) EMA statement on ongoing review of GLP-1 receptor agonists. Available from https://www.ema.europa.eu/en/news/ema-statement-ongoing-review-glp-1-receptor-agonists. Accessed 29 January 2024

[12] Ruder K (2023) As semaglutide’s popularity soars, rare but serious adverse effects are emerging. JAMA 330(22):2140–2142. 10.1001/jama.2023.16620 37966850 10.1001/jama.2023.16620

[13] Cohen D (2023) GLP-1 receptor agonists: European drug regulator asks makers for evidence of self-harm. BMJ 383:2906. 10.1136/bmj.p2906 38084503 10.1136/bmj.p2906

[14] U.S. Food and Drug Administration (2024) Update on FDA’s ongoing evaluation of reports of suicidal thoughts or actions in patients taking a certain type of medicines approved for type 2 diabetes and obesity. Preliminary evaluation does not suggest a causal link. Available from https://www.fda.gov/drugs/drug-safety-and-availability/update-fdas-ongoing-evaluation-reports-suicidal-thoughts-or-actions-patients-taking-certain-type. Accessed 29 January 2024

[15] García-Sempere A, Orrico-Sánchez A, Muñoz-Quiles C et al (2020) Data resource profile: the Valencia health system integrated database (VID). Int J Epidemiol 49(3):740–741e. 10.1093/ije/dyz266 31977043 10.1093/ije/dyz266 PMC7394961

[16] Valencia Regional Government (2021) Guideline for the prescription and prior authorization of antidiabetic drugs. Available from https://www.imeval.org/wp-content/uploads/2015/12/GUIA-PARA-LA-PRESCRIPCION-Y-VISADO-DE-ANTIDIABETICOS-17nov2021.pdf [report in Spanish]. Accessed 6 April 2024

[17] Austin PC (2011) An introduction to propensity score methods for reducing the effects of confounding in observational studies. Multivar Behav Res 46(3):399–424. 10.1080/00273171.2011.568786 10.1080/00273171.2011.568786 PMC3144483 21818162

[18] Austin PC (2009) Balance diagnostics for comparing the distribution of baseline covariates between treatment groups in propensity-score matched samples. Stat Med 28(25):3083–3107. 10.1002/sim.3697 19757444 10.1002/sim.3697 PMC3472075

[19] Luppino FS, de Wit LM, Bouvy PF et al (2010) Overweight, obesity, and depression: a systematic review and meta-analysis of longitudinal studies. Arch Gen Psychiatry 67(3):220–229. 10.1001/archgenpsychiatry.2010.2 20194822 10.1001/archgenpsychiatry.2010.2

[20] McIntyre RS (2024) Glucagon-like peptide-1 receptor agonists (GLP-1 RAs) and suicidality: what do we know and future vistas. Expert Opin Drug Saf 26:1–4. 10.1080/14740338.2024.2335215 10.1080/14740338.2024.2335215 38520274

[21] Klinitzke G, Steinig J, Blüher M, Kersting A, Wagner B (2013) Obesity and suicide risk in adults—a systematic review. J Affect Disord 145(3):277–284. 10.1016/j.jad.2012.07.010 22871535 10.1016/j.jad.2012.07.010

[22] Lau D, Gamble JM (2024) Suicidality among users of glucagon-like peptide-1 receptor agonists: an emerging signal? Diabetes Obes Metab 26(4):1150–1156. 10.1111/dom.15459 38229461 10.1111/dom.15459

[23] Astreboff AM, Aronne LJ, Ahmad NN et al (2022) Tirzepatide once weekly for the treatment of obesity. N Engl J Med 387(3):205–216. 10.1056/NEJMoa2206038 35658024 10.1056/NEJMoa2206038

[24] Lincoff AM, Brown-Frandsen K, Colhoun HM et al (2023) Semaglutide and cardiovascular outcomes in obesity without diabetes. N Engl J Med 389(24):2221–2232. 10.1056/NEJMoa2307563 37952131 10.1056/NEJMoa2307563

[25] O’Neil PM, Aroda VR, Astrup A et al (2017) Neuropsychiatric safety with liraglutide 3.0 mg for weight management: results from randomized controlled phase 2 and 3a trials. Diabetes Obes Metab 19(11):1529–1536. 10.1111/dom.12963 28386912 10.1111/dom.12963 PMC5655710

[26] Garvey WT, Batterham RL, Bhatta M et al (2022) Two-year effects of semaglutide in adults with overweight or obesity: the STEP 5 trial. Nat Med 28(10):2083–2091. 10.1038/s41591-022-02026-4 36216945 10.1038/s41591-022-02026-4 PMC9556320

[27] McIntyre RS, Mansur RB, Rosenblat JD, Kwan ATH (2024) The association between glucagon-like peptide-1 receptor agonists (GLP-1 RAs) and suicidality: reports to the Food and Drug Administration Adverse Event Reporting System (FAERS). Expert Opin Drug Saf 23(1):47–55. 10.1080/14740338.2023.2295397 38087976 10.1080/14740338.2023.2295397

[28] Guirguis A, Chiappini S, Papanti PGD et al (2024) Exploring the association between suicidal thoughts, self-injury, and GLP-1 receptor agonists in weight loss treatments: insights from pharmacovigilance measures and unmasking analysis. Eur Neuropsychopharmacol 82:82–91. 10.1016/j.euroneuro.2024.02.003 38508100 10.1016/j.euroneuro.2024.02.003

[29] Chen C, Zhou R, Fu F, Xiao J (2023) Postmarket safety profile of suicide/self-injury for GLP-1 receptor agonist: a real-world pharmacovigilance analysis. Eur Psychiatry 66(1):e99. 10.1192/j.eurpsy.2023.2474 38031404 10.1192/j.eurpsy.2023.2474 PMC10755578

[30] Zhou J, Zheng Y, Xu B et al (2024) Exploration of the potential association between GLP-1 receptor agonists and suicidal or self-injurious behaviors: a pharmacovigilance study based on the FDA Adverse Event Reporting System database. BMC Med 22(1):65. 10.1186/s12916-024-03274-6 38355513 10.1186/s12916-024-03274-6 PMC10865629

[31] EMA requests more data from GLP-1 drugmakers in suicidal ideation, self-harm probe (2023) Available from https://www.biospace.com/article/ema-requests-more-data-from-glp-1-drugmakers-in-suicidal-ideation-self-harm-probe/. Accessed 7 April 2024

[32] Ruggiero R, Mascolo A, Spezzaferri A, Carpentieri C, Torella D, Sportiello L (2024) Glucagon-like peptide-1 receptor agonists and suicidal ideation: analysis of real-word data collected in the European pharmacovigilance database. Pharmaceuticals (Basel) 17(2):147. 10.3390/ph17020147 38399362 10.3390/ph17020147 PMC10892952

[33] Wang W, Volkow ND, Berger NA, Davis PB, Kaelber DC, Xu R (2024) Association of semaglutide with risk of suicidal ideation in a real-world cohort. Nat Med 30(1):168–176. 10.1038/s41591-023-02672-2 38182782 10.1038/s41591-023-02672-2 PMC11034947

[34] Nassar M, Misra A, Bloomgarden Z (2024) Impact of treatment with GLP-1RAs on suicide attempts in adults persons with type 2 diabetes: a retrospective comparative effectiveness study based on a global TriNetX health research database. J Diabetes 16(3):e13547. 10.1111/1753-0407.13547 38501220 10.1111/1753-0407.13547 PMC10949079

[35] Simon GE, Shortreed SM, Boggs JM et al (2022) Accuracy of ICD-10-CM encounter diagnoses from health records for identifying self-harm events. J Am Med Inform Assoc 29(12):2023–2031. 10.1093/jamia/ocac144 36018725 10.1093/jamia/ocac144 PMC9667165

[36] Swain RS, Taylor LG, Braver ER, Liu W, Pinheiro SP, Mosholder AD (2019) A systematic review of validated suicide outcome classification in observational studies. Int J Epidemiol 48(5):1636–1649. 10.1093/ije/dyz038 30907424 10.1093/ije/dyz038

[37] Porta M (2014) A dictionary of epidemiology, 6th edn. Oxford University Press/International Epidemiological Association, Oxford, p 343. 10.1093/acref/9780199976720.001.0001

[38] Meissner WG, Remy P, Giordana C et al (2024) Trial of lixisenatide in early Parkinson’s disease. N Engl J Med 390(13):1176–1185. 10.1056/NEJMoa2312323 38598572 10.1056/NEJMoa2312323

[39] European Medicines Agency (2024) Meeting highlights from the Pharmacovigilance Risk Assessment Committee (PRAC) 8-11 April 2024. GLP-1 receptor agonists: available evidence not supporting link with suicidal and self-injurious thoughts and actions. Available from https://www.ema.europa.eu/en/news/meeting-highlights-pharmacovigilance-risk-assessment-committee-prac-8-11-april-2024. Accessed 23 July 2024

