# Supplementary material for: Association of glucagon-like peptide-1 receptor agonists with suicidal ideation and self-injury in individuals with diabetes and obesity: a propensity-weighted, population-based cohort study
Source: Diabetologia. 2024 Aug 6;67(11):2471–80. doi: 10.1007/s00125-024-06243-z (PMC11519213; doi:10.1007/s00125-024-06243-z)

Association of glucagon-like peptide-1 receptor agonists with suicidal ideation and self-injury in individuals with diabetes and obesity: a propensity-weighted, population-based cohort study.

Electronic Supplementary Material

Index to the Electronic Supplementary Material

ESM Table 1 International Classification of Diseases, Ninth and Tenth Revision (ICD-9 and ICD-10) codes for comorbidities, exclusion criteria, lifestyle variables and outcomes.

ESM Table 2. Anatomical Therapeutic Chemical classification for drugs included in the study.

ESM Table 3. Code employed for analysis.

ESM Fig. 1. Forest plot for stratified analyses per sex, obesity, depression, sleep disorders and anxiety, plotted in logarithmic scale. P values for interaction and hazard ratios (95%CI) for each group are shown.

ESM Table 1. International Classification of Diseases, Ninth and Tenth Revision (ICD-9 and ICD-10) codes for comorbidities, exclusion criteria, lifestyle variables and outcomes.

Heart Failure	ICD9<br>428.xx, 398.91, 402.01, 402.11, 402.91, 404.01, 404.11, 404.03, 404.13, 404.91, 404.93, 425.4x<br>ICD10<br>I50.x, I11.0, I13.0, I13.2, I42.0
Dementia	ICD9<br>290.xx, 294.xx, 330.xx, 331.xx<br>ICD10<br>F00.x- F03.x, G30.x, G31.x
Hypertension	ICD9<br>401.xx-405.xx, 437.2<br>ICD10<br>I10.x-I13.x, I15.x, I67.4
Liver disease	ICD9<br>070.0, 070.2x, 070.4x, 070.6x, 070.71, 570.xx-573.xx,782.4x,789.1x,789.5x,790.4x,790.5x,794.8x<br>ICD10<br>B19.11, B15.0x, B16.0, B16.2, B19.0, I85.x, K70.x-K77.x, R17.x,R18.x,R74.x,R94.5,Z94.4
Renal disease	ICD9<br>403.x, 404.x, 580.x-589.x,590.1, 753.x, 792.5,V42.0,V45.1,V56.x<br>ICD10<br>I12.x, I13.x, N00.x-N05.x, N07.x, N11.x, N14.x, N17.x-N19.x, Q61.x,Z49.x,Z94.0,Z91.15, Z99.2
Coronary heart disease	ICD9<br>410.xx-414.xx<br>ICD10<br>I20.x-I25.x
Chronic Obstructive Pulmonary Disease	ICD9<br>491.xx,492.xx,496.xx,493.20,493.21,493.22<br>ICD10 diagnosis<br>J41.x- J44.x
Malignancies	ICD9<br>140.xx-208.xx<br>ICD10<br>C00.x-C96.x
Obesity	ICD9<br>278.8, 278.00, 278.01, 278.03,E66.8, E66.9, V85.3,V85.4<br>ICD10<br>Z68.3,Z68.4
Suicidal ideation or self-injury	ICD9<br>E95.x,V62.84, 300.9<br>ICD10<br>T36.92X , T37.92X , T39.92X , T41.42X , T42.72X , T43.92X , T45.92X, T47.92X , T49.92X , T51.92X , T52.92X , T53.92X , T54.92X , T56.92X , T57.92X ,T59.92X , T60.92X , T61.92X , T62.92X , T63.92X , T65.92X , X71.x-X83.x , T58.02X , T58.03X ,

	T58.04X , T58.05X , T58.06X , T58.07X , T58.08X, T58.09X , T58.10X , T58.11X , T58.12X, T61.02X , T61.03X , T61.04X, T61.05X, T61.06X , T61.07X , T61.08X), T61.09X , T61.10X , T61.11X , T61.12X, T64.02X , T64.03X , T64.04X , T64.05X , T64.06X , T64.07X , T64.08X, T64.09X , T64.10X , T64.11X , T64.12X, R45.851 T14.91X, Z91.5,T14.91,<br>T36–T65 with the 6th character of 2 (except for T36.9, T37.9, T39.9, T41.4, T42.7, T43.9, T45.9, T47.9, T49.9, T51.9, T52.9, T53.9, T54.9, T56.9, T57.9, T58.0, T58.1, T58.9, T59.9, T60.9, T61.0, T61.1, T61.9, T62.9, T63.9, T64.0, T64.8, and T65.9, which are included if the 5th character is 2)
Anxiety	ICD9<br>300.x, 308.x<br>ICD10<br>F40.x-F48.x
Depression	ICD9<br>296.2x. 296.3x, 298.0, 300.4x, 301.12, 311.xx<br>ICD10<br>F32.x-F33.x; F34.1
Alcohol use	ICD9<br>291.xx, 303.xx, 305.0x, 571.0x, 571.1x, 571.2x, 571.3x, 357.5x, 425.5x, E860.0x<br>ICD10<br>F10.x; K70.x; G62.1; I42.6
Tobacco use	ICD9<br>305.1,989.84,V15.82<br>ICD10<br>F17.x,T65.2
Drug use	ICD9<br>292.xx, 304.xx, 305.2x-305.9x<br>ICD10<br>F11.x-F16.x; F18.x-F19.x
Personality disorders	ICD9<br>301.xx<br>ICD10<br>F60.x
Sleep disorders	ICD9<br>307.4x, 327.xx, 780.5x, 347.xx<br>ICD10<br>G47.x; F51.x; Z72.82x
Psychotic disorders	ICD9<br>290.x-299.x<br>ICD10<br>F20.x-F29.x
Other psychiatric disorders	Bipolar disorders<br>ICD9<br>296.0x, 296.1x, 296.4x, 296.5x, 296.6x, 296.7x, 296.8x, 296.99<br>ICD10<br>F31.x<br>Disturbance of conduct<br>ICD9

309.x; 312.x
ICD10
F91.x
Hyperkinetic syndrome of childhood
314.xx
ICD10
F90.x

ESM Table 2. Anatomical Therapeutic Chemical classification of study drugs

Sodium-glucose cotransporter 2 inhibitors (SGLT-2i)	A10BK. Combination with metformin: A10BD15, A10BD16, A10BD20, A10BD23
Glucagon-like peptide 1 receptor (GLP-1RA) agonists	A10BJ, A10AE54, A10AE56, A10BX04, A10BX07, A10BX10, A10BX13,A10BX14
Metformin monotherapy	A10BA02

ESM Table 3. Code employed for analysis.

```
***********************************
*PRINCIPAL ANALYSIS - PER PROTOCOL*
***********************************
use "Q:\ANTIDIABETICOS_suicide\3-ANALISIS\FINAL_CONJUNTO\cohorte_final_censura_sinunderyover.dta",clear


*COVARIATES SETUP
global cov_prop_scores sex  i.income  i.cat_age c_heart_f c_dementia c_hyperte c_liv ///
c_kidney c_ethanol c_depression c_chd c_codp c_smoke c_sleepdisord c_anxiety c_otherabuse c_persondis c_otherpsych c_psychotic c_cancer c_suicide i.imc_bis


*GENERATE IPTW
logit clas_bi $cov_prop_scores
predict ps_clas_bi
propwt clas_bi ps_clas_bi, ipt smr


*CALCULATE P FOR INTERACTION BETWEEN GROUPING VARIABLE AND SOME COVARIATES
logit o_suicide clas_bi  sex b(first).clas_bi#b(first).sex
logit o_suicide c.cat_age##i.clas_bi
logit o_suicide c.income##i.clas_bi
logit o_suicide c.imc_bis##i.clas_bi
logit o_suicide clas_bi  c_depression b(first).clas_bi#b(first).c_depression
logit o_suicide clas_bi  c_sleepdisord b(first).clas_bi#b(first).c_sleepdisord
logit o_suicide clas_bi  c_anxiety b(first).clas_bi#b(first).c_anxiety


*COX MODEL
*Define censoring date and fix outcome variable
replace o_suicide=0 if censor_date<suicide_date & suicide_date!=.
replace censor_date=suicide_date if suicide_date!=. & censor_date>suicide_date & o_suicide==1


stset censor_date [pweight=ipt_wt], failure(o_suicide) origin(index_date) id(sip) scale(365.25)
stdes
```

```
stcox clas_bi $cov_prop_scores


*********************

*WITHOUT UNKNOWN BMI*

*********************


use "Q:\ANTIDIABETICOS_suicide\3-
ANALISIS\FINAL_CONJUNTO\cohorte_final_censura_sinunderyover.dta",clear


*DROP THE PATIENTS WITH UNKNOWN BMI

drop if imc_bis==9


*COVARIATES SETUP

global cov_prop_scores sex  i.income  i.cat_age c_heart_f c_dementia c_hyperte c_liv ///

c_kidney c_ethanol c_depression c_chd c_codp c_smoke c_sleepdisord c_anxiety c_otherabuse
c_persondis c_otherpsych c_psychotic c_cancer c_suicide i.imc_bis


*GENERATE IPTW

logit clas_bi $cov_prop_scores

predict ps_clas_bi

propwt clas_bi ps_clas_bi, ipt smr


*COX MODEL

*Define censoring date and fix outcome variable

replace o_suicide=0 if censor_date<suicide_date & suicide_date!=.

replace censor_date=suicide_date if suicide_date!=. & censor_date>suicide_date & o_suicide==1


stset censor_date [pweight=ipt_wt], failure(o_suicide) origin(index_date) id(sip) scale(365.25)

stdes

stcox clas_bi $cov_prop_scores


*********************

*STRATIFIED ANALYSIS*

*********************
```

```
*SEX

stcox clas_bi if sex==1

stcox clas_bi if sex==2


*OBESITY

stcox clas_bi if imc_bis==3

stcox clas_bi if imc_bis==4

stcox clas_bi if imc_bis==5


*DEPRESSION

stcox clas_bi if c_depression==0

stcox clas_bi if c_depression==1


*SLEEP DIS

stcox clas_bi if c_sleepdisord==0

stcox clas_bi if c_sleepdisord==1


*ANXIETY

stcox clas_bi if c_anxiety==0

stcox clas_bi if c_anxiety==1


*GLOBAL

stcox clas_bi


********************

*INTENTION TO TREAT*

********************


use "Q:\ANTIDIABETICOS_suicide\3-
ANALISIS\FINAL_CONJUNTO\cohorte_final_censura_sinunderyover.dta",clear


*COVARIATE SETUP

global cov_prop_scores sex  i.income  i.cat_age c_heart_f c_dementia c_hyperte c_liv ///
```

```
c_kidney c_ethanol c_depression c_chd c_codp c_smoke c_sleepdisord c_anxiety c_otherabuse
c_persondis c_otherpsych c_psychotic c_cancer c_suicide i.imc_bis


*GENERATE IPTW
logit clas_bi $cov_prop_scores
predict ps_clas_bi
propwt clas_bi ps_clas_bi, ipt smr


*COX MODEL
*Define censoring date and fix outcome variable
drop censor_date
gen censor_date=mdy(12,31,2021)
replace censor_date=def_date if def_date<=censor_date & def_date>index_date
replace o_suicide=0 if censor_date<suicide_date & suicide_date!=.
replace censor_date=suicide_date if suicide_date!=. & censor_date>suicide_date & o_suicide==1


stset censor_date [pweight=ipt_wt], failure(o_suicide) origin(index_date) id(sip) scale(365.25)
stdes
stcox clas_bi  $cov_prop_scores


**********************************
*MULTIPLE IMPUTATION PER PROTOCOL*
**********************************


use "Q:\ANTIDIABETICOS_suicide\3-
ANALISIS\FINAL_CONJUNTO\cohorte_final_censura_sinunderyover.dta",clear


*RECODE TO MISSING
recode imc_bis (9=.)


*IMPUTATION OF BMI BY ICE PROTOCOL
ice imc_bis clas_bi sex  i.income  i.cat_edad c_heart_f c_dementia c_hyperte c_liv ///
c_kidney c_ethanol c_depression c_chd c_codp c_smoke c_sleepdisord c_anxiety c_otherabuse
c_persondis c_otherpsych c_psychotic c_cancer c_suicidio o_suicidio_periodo,
```

```
cmd(imc_bis:ologit)seed(1001) m(5) saving("Q:\ANTIDIABETICOS_SUICIDIO\3-ANALISIS\multiple.dta",replace)

use "Q:\ANTIDIABETICOS_SUICIDIO\3-ANALISIS\multiple.dta",replace

mi import ice


*COVARIATE SET UP

global cov_prop_scores sex  i.income  i.cat_age c_heart_f c_dementia c_hyperte c_liv ///

c_kidney c_ethanol c_depression c_chd c_codp c_smoke c_sleepdisord c_anxiety c_otherabuse c_persondis c_otherpsych c_psychotic c_cancer c_suicide i.imc_bis


*GENERATE IPTW

mi estimate,  saving("Q:\ANTIDIABETICOS_SUICIDIO\3-ANALISIS\imput_imc_ice.ster",replace): logit clas_bi $cov_prop_scores

mi predict ps_clas_bi using "Q:\ANTIDIABETICOS_SUICIDIO\3-ANALISIS\imput_imc_ice.ster"

quietly mi xeq: generate ps_clas_bi_t = invlogit(ps_clas_bi)


gen iptw=1/ps_clas_bi_t if clas_bi==1

replace iptw = 1 / (1 - ps_clas_bi_t) if clas_bi==0


sort sip iptw

by sip: replace iptw=iptw[1] if iptw==.


*COX MODEL

*Define censoring date and fix outcome variable

replace o_suicide=0 if censor_date<suicide_date & suicide_date!=.

replace censor_date=suicide_date if suicide_date!=. & censor_date>suicide_date & o_suicide==1

mi stset censor_date [pweight=iptw] , failure(o_suicide) origin(index_date) id(sip) scale(365.25)

mi estimate,hr : stcox clas_bi  $cov_prop_scores
```

ESM Fig. 1. Forest plot for stratified analyses per sex, obesity, depression, sleep disorders and anxiety, plotted in logarithmic scale. P values for interaction, number of events and hazard ratios for each group are shown.

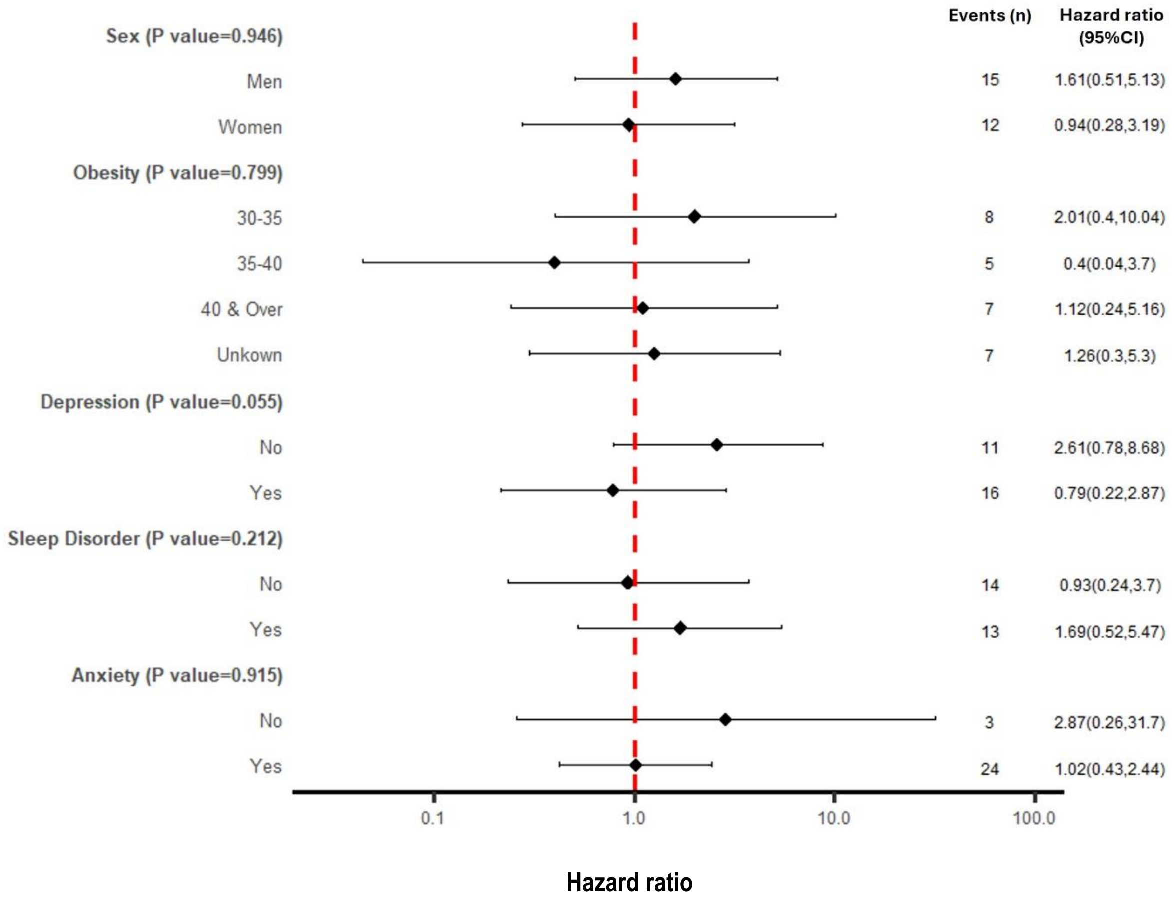

Supplement: Supplementary file 1 — ESM 1 (PDF 258 KB) [file 125_2024_6243_MOESM1_ESM.pdf]
